# Genetically predicted blood metabolites mediate the association between immune cell characteristics and urolithiasis: A Mendelian randomization study and mediation analysis

**DOI:** 10.1016/j.gendis.2025.101547

**Published:** 2025-01-28

**Authors:** Chengcheng Wei, Jiatai He, Jun Wen, Shunyao Wang, Mengjia Shi, Juan Hu, Huanhuan Tan, Jinjun Guo, Xiaosong Li

**Affiliations:** a Department of Urology, The First Affiliated Hospital of Chongqing Medical University, Chongqing 400016, China; b Department of Urology, Union Hospital, Tongji Medical College, Huazhong University of Science and Technology, Wuhan, Hubei 430074, China; c Department of Respiratory and Critical Care Medicine, The First Affiliated Hospital of Chongqing Medical University, Chongqing Medical University, Chongqing 400016, China; d Clinical Molecular Medicine Testing Center, The First Affiliated Hospital of Chongqing Medical University, Chongqing 400016, China; e Department of Clinical Laboratory Medicine Suining Central Hospital, Suining, Sichuan 629000, China; f Reproductive Medicine Center, The First Affiliated Hospital of Chongqing Medical University, Chongqing 400016, China; g Bishan Hospital of Chongqing, Bishan Hospital of Chongqing Medical University, Chongqing 402760, China; h Western Institute of Digital-Intelligent Medicine, Chongqing 401329, China

**Keywords:** Blood metabolites, Immunity, Mediation analysis, Mendelian randomization study, Urolithiasis

## Abstract

Urolithiasis, a disease characterized by the formation of urinary stones, is influenced by immune system dysregulation and metabolic factors. This study investigated the interplay between specific immune cell characteristics and blood metabolites in urolithiasis based on Mendelian randomization. We further explored the potential mediating effects of genetically predicted blood metabolites based on mediation analysis. We employed a two-sample Mendelian randomization analysis to examine the association between immune cell properties, blood metabolites, and urolithiasis risk. Genetic instruments for immune cell characteristics and blood metabolites were used to assess causal relationships and mediating pathways. Our results indicate that 10 immune cell characteristics had a unidirectional causal association with urolithiasis risk. We also detected 13 blood metabolites associated with urolithiasis. We identified 4 pathways through which genetically predicted blood metabolites partly mediated the association between specific immune cell characteristics and urolithiasis risk. This suggests potential mechanistic links where altered blood metabolites may play a role in developing urolithiasis through immune system modulation. This Mendelian randomization study highlights the complex relationship between immune responses, blood metabolites, and urolithiasis. The findings underscore the importance of considering both immune cell features and metabolic factors in understanding the pathogenesis of urolithiasis, offering insights into novel therapeutic targets and diagnostic strategies for this disorder.

## Introduction

As an intricate disease influenced by genetic and environmental factors, urolithiasis is highly prevalent worldwide and affects 10%–15% of the world's population, whose prevalence indicates a growing trend and ranges from 5% to 9%, especially in Europe.^1^, ^2^, ^3^ In addition to significantly lowering patients' quality of life, the high recurrence rate of urolithiasis raises the risk of fractures, renal insufficiency, and cardiovascular disorders, which adds to their disease burden.^4^ A systematic review indicates that urolithiasis puts a significant physical and psychological strain on the stone formers in addition to their financial burden, and it is projected that by 2030, the annual cost of treating urolithiasis in the US will rise by $1.24 billion per year.^5^^,^^6^ Numerous factors, such as genetics, environment, diet, obesity, gut microbiota, infection *etc*., might influence the development of urolithiasis.^7^, ^8^, ^9^ Exploring the correlation and mechanism between pathogenic factors and the occurrence of urolithiasis is an important means of preventing and monitoring urolithiasis.

Previous studies have shown that increased gene expression linked to immunological, complement, and inflammatory pathways in renal tissue is linked to the development of urolithiasis.^10^ Calcium oxalate (CaOx) crystals have been shown to promote urolithiasis formation by causing M1 macrophage polarization and triggering monocyte inflammatory response.^11^ The animal model of urolithiasis constructed by Kumar et al found that hydroxy-l-proline induced an increase in markers of inflammation and changes in the renal immune system, revealing the possible role of immune cells in the formation of urolithiasis.^12^ In reality, inflammation in urolithiasis disease can occur either as a downstream consequence or as an upstream pathogenic component. Despite the information presented above, further study is required to completely comprehend the immune system and immunity in urolithiasis.

Recent studies have shown that urolithiasis is increasingly seen as a chronic metabolic condition.^13^^,^^14^ Most non-infectious stones include CaOx, the most prevalent chemical composition,^15^^,^^16^ whose formation is closely related to high urinary calcium (hypercalciuria) and oxalate (hyperoxaluria).^17^^,^^18^ However, there is evidence to suggest that some metabolites have a protective effect on the formation of stones. Zhang et al found that succinate may play a protective role against stone formation by reducing inflammation, preventing cell adhesion, and promoting osteogenic differentiation.^13^ The impact of metabolism on urolithiasis is still controversial and multifaceted.^19^ Therefore, studies on how urolithiasis forms based on metabolism may be very valuable for treating this illness.

In this study, we used Mendelian randomization (MR) to investigate if immune cell features are risk factors for urolithiasis. MR is a popular genetic epidemiology method that studies the possible causative relationship between risk factors and urolithiasis using single nucleotide polymorphisms (SNPs) as instrumental variables (IVs). Because alleles are randomly divided during meiosis, confounding variables (such as behavior and environmental exposure) are less likely to influence MR. Because genetic variation occurs before illness and cannot be reversed, MR may also lessen the reverse causation bias inherent in observational research.^20^^,^^21^ Therefore, we used MR mediation to look for putative mediators between immune cell features and urolithiasis to better understand the pathophysiological modulators of immune cell–urolithiasis interactions.

## Methods

### Study design

Figure 1 shows the general arrangement of this study. The methods used in the study met the requirements of STROBE-MR, or Strengthening the Reporting of Observational Studies in Epidemiology-Mendelian Randomization. In step 1, we conducted an analysis to examine the causative impacts of 731 immune cell characteristics on urolithiasis. In step 2, we investigated the causal effects of 1400 blood metabolites on urolithiasis. Finally, in step 3, we employed mediation analysis to investigate the role of blood metabolites in the pathway linking immune cell characteristics to urolithiasis. SNPs were designated as instrumental variables (IVs). MR relied on three fundamental assumptions: i) The IVs had a strong correlation with the exposure factors; ii) The IVs were not correlated with any confounding factors; iii) The IVs did not have a direct impact on the result, and their influence on the outcome was solely through exposure.

**Figure 1:**
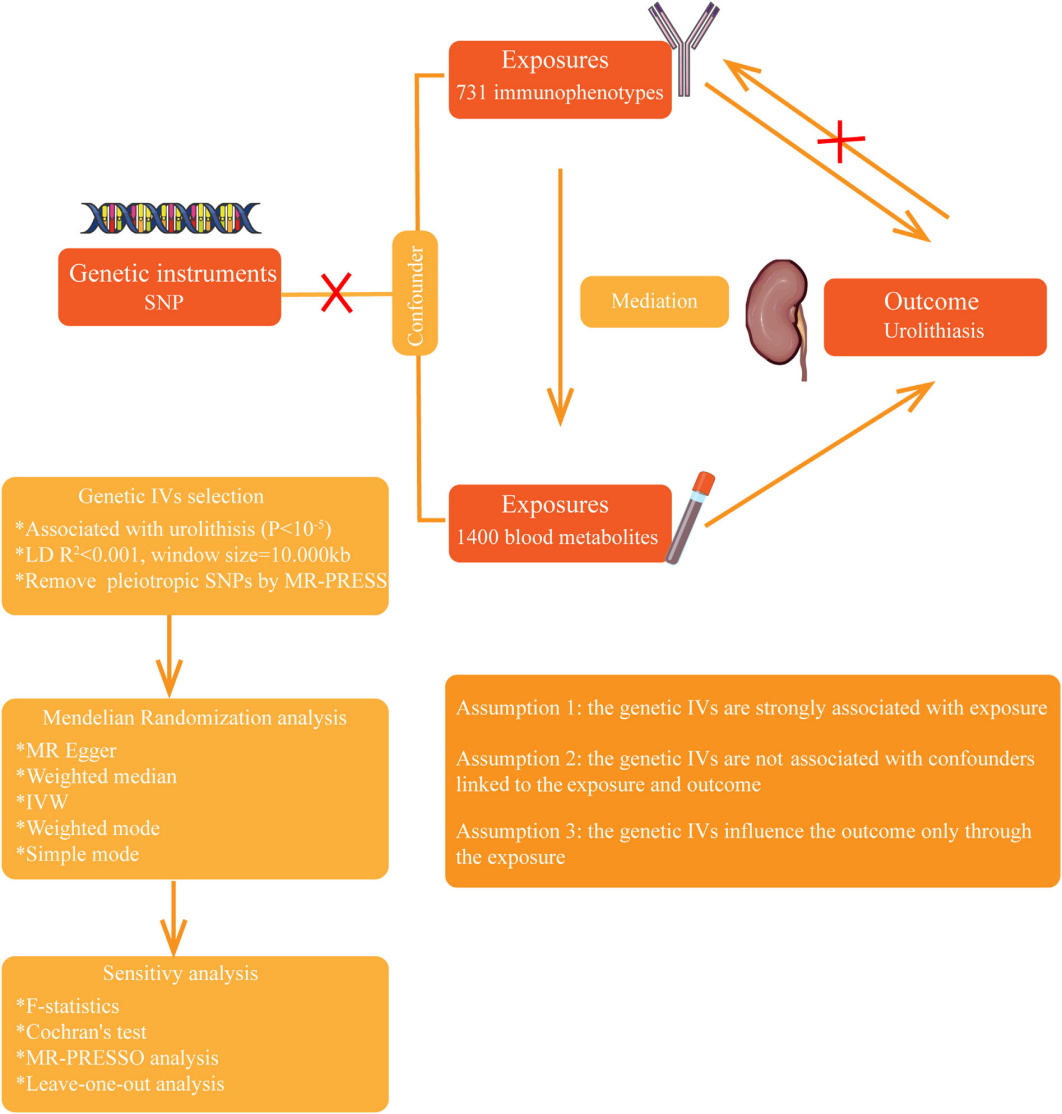
Flowchart of study.

### Genetic instruments for metabolites and immune cell characteristics

The genome-wide association study (GWAS) summary data for each immunological feature may be accessed publicly from the GWAS Catalog. The accession numbers range from GCST0001391 to GCST0002121.^22^ There was a total of 731 immunophenotypes included in the study. These included 389 median fluorescence intensities, which indicate surface antigen levels, 118 absolute cell counts, 32 morphological traits, and 192 relative cell counts. B cells, conventional dendritic cells (CDCs), mature T cells, monocytes, myeloid cells, TBNK (T cells, B cells, natural killer cells), and Treg panels make up the immune traits including median fluorescence intensity, relative cell count, absolute cell count. However, the TBNK and CDC panels are part of the immune trait, morphological parameters. The initial GWAS on immunological variables included data from 3757 people of European descent, with no cohorts that shared participants. Around 22 million SNPs were genotyped using high-density arrays and then imputed using a Sardinian sequence-based reference panel.^23^ The GWAS Catalog (https://www.ebi.ac.uk/gwas/) contains summary data on different metabolites from several GWAS studies. The accession numbers for European GWASs are GCST90199621–90201020. The data provided encompasses a comprehensive set of 1400 blood metabolites. We conducted a search utilizing the GWAS summary data (http://gwas.mrcieu.ac.uk), which encompasses an extensive compilation of summary statistics from various GWASs. We used publicly accessible summary statistic information from a GWAS on persons of European descent who were diagnosed with urolithiasis. The specific dataset we employed is ebi-a-GCST90018935, which includes a total of 488,346 individuals. Among them, there were 6223 cases and 482,123 control cases.

### Selection of instrumental variables

Based on current research, the importance level of IVs for each immunological characteristic was established at 1 × 10^−5^. The PLINK program (version 1.90) was utilized to perform the clumping technique on these SNPs. The SNPs were pruned based on a linkage disequilibrium R^2^ threshold of less than 0.001 at a distance of 1000 kb. The linkage disequilibrium R^2^ values were obtained using the 1000 Genomes Projects as a reference panel. In the case of urolithiasis, we modified the significance threshold to 5 × 10^−8^. The proportion of variance in phenotypes explained (PVE) by available genotypes or SNP heritability, and F statistics were computed for each IV to assess the strength of the IV and prevent any potential bias caused by weak instruments.

### Statistical analysis

To evaluate the causative association between 1400 blood metabolites and urolithiasis, as well as 731 immunophenotypes, we mainly used the “TwoSampleMR” software (version 4.2.1) to perform MR-Egger, weighted median, weighted mode, inverse variance weighting (IVW), and simple mode techniques. The Cochran's Q statistic and its accompanying *p*-values were utilized to assess the heterogeneity among the chosen IVs. If the null hypothesis is rejected, the fixed-effects IVW method was replaced by the random-effects IVW method. To account for the impact of horizontal pleiotropy, a widely employed technique called MR-Egger was utilized. This approach detects the existence of horizontal pleiotropy if its intercept term is shown to be statistically significant. Considering that the majority of our GWAS summary results are derived from European populations, it is imperative to acknowledge the potential for sample overlap.^24^ The mediation analysis included immune cell features and blood metabolites that had substantial causal impacts on urolithiasis, as determined by the two-sample study. We looked at any possible links between blood metabolites and immune cell properties. If such a relationship exists, we would do a mediation analysis to examine if blood metabolites serve as mediators in the pathway from immune cell characteristics to urolithiasis. We conducted Cochran's Q test to assess the heterogeneity of each SNP. Additionally, we created scatter plots to depict the relationships between SNPs and both exposure and outcome variables, to present the findings of MR analysis. A leave-one-out analysis was conducted to analyze the impact of each SNP on the results. This involved progressively eliminating each SNP and using the IVW approach to the remaining SNPs to evaluate the possible influence of a specific variation on the estimations. Furthermore, we employed MR-PRESSO and MR-Egger regression techniques to examine the possible impact of horizontal pleiotropy. The MR-PRESSO method was employed to identify and eliminate significant outliers, hence correcting the horizontal plural effect. The studies were conducted using the R (version 4.2.1) statistical program. The multiplicity tests utilized the “MR_PRESSO” program.

## Results

### Screening the causal effect of immune cell characteristics on urolithiasis

Table S1 provides SNP-related information. As shown in Figure 2 and Table S2, MR analysis was conducted on 731 immune cell signatures and urolithiasis. The heatmap presented the results of IVW *p*-values < 0.1. Then, we filtered the above results for inconformity using the following standards: i) IVW *p*-value < 0.01; ii) Five MR models had consistent direction; iii) Pleiotropy *p*-value < 0.05 (Table S3). There were 10 suggestive immunophenotypes identified, of which 3 were in the B cell panel, 2 in the Treg panel, 1 in the maturation stage panel, 3 in the CDC panel, and 1 in the TBNK panel (Table S4). For B cell panel, three traits were linked with increased urolithiasis risk: IgD-CD24-%lymphocyte (IVW: odds ratio/OR = 1.054; 95% confidence interval/CI: 1.019, 1.090; *p* = 0.002), CD24 on transitional B cells (IVW: OR = 1.068; 95% CI: 1.020, 1.117; *p* = 0.005), and CD25 on IgD^+^ CD38-naïve (IVW: OR = 1.041; 95% CI: 1.010, 1.073; *p* = 0.009). For the Treg cell panel, two traits were linked with increased urolithiasis risk: CD4 Treg %T cell (IVW: OR = 1.060; 95% CI: 1.015, 1.108; *p* = 0.009) and activated and resting Treg AC (IVW: OR = 1.058; 95% CI: 1.018, 1.100; *p* = 0.004). In the maturation stages of the T cell panel, CD3 on TD CD4^+^ was linked with decreased urolithiasis risk (IVW: OR = 0.963; 95% CI: 0.939, 0.986; *p* = 0.002). In the CDC panel, CD11c on myeloid DC (IVW: OR = 1.045; 95% CI: 1.013, 1.077; *p* = 0.006) was linked with increased urolithiasis risk. Two traits were linked with decreased urolithiasis risk: HLA DR on plasmacytoid DC (IVW: OR = 0.972; 95% CI: 0.955, 0.988; *p* = 0.001) and HLA DR on DC (IVW: OR = 0.970; 95% CI: 0.949, 0.991; *p* = 0.006). In the TBNK panel, HLA DR on B cells was linked with decreased urolithiasis risk (IVW: OR = 0.964; 95% CI: 0.940, 0.989; *p* = 0.004). Remarkably, all of the B cell traits and Tregs traits were risk factors, including IgD-CD24-% lymphocyte, CD24 on transitional B cells, CD25 on IgD^+^ CD38-naïve, CD4 Treg %T cell, and activated and resting Treg AC (Fig. 3A and Table S5).

**Figure 2:**
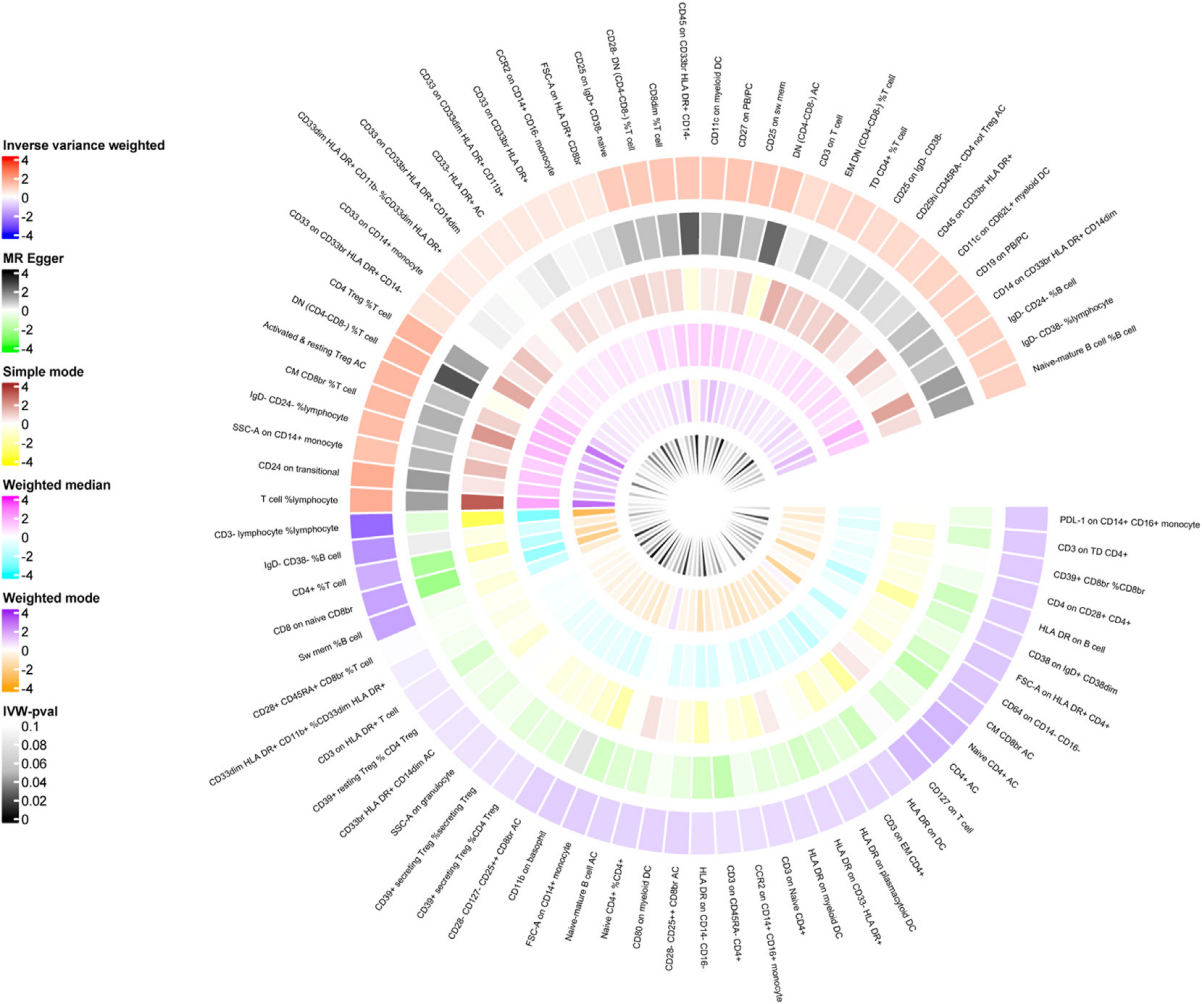
The heatmap depicting the association between immune cell characteristics and urolithiasis. It shows all the results with IVW *p*-value < 0.01. The outer circle represents the names of immune cell characteristics, while the inner circle uses different colors to indicate the odds ratio of five Mendelian randomization models.

**Figure 3:**
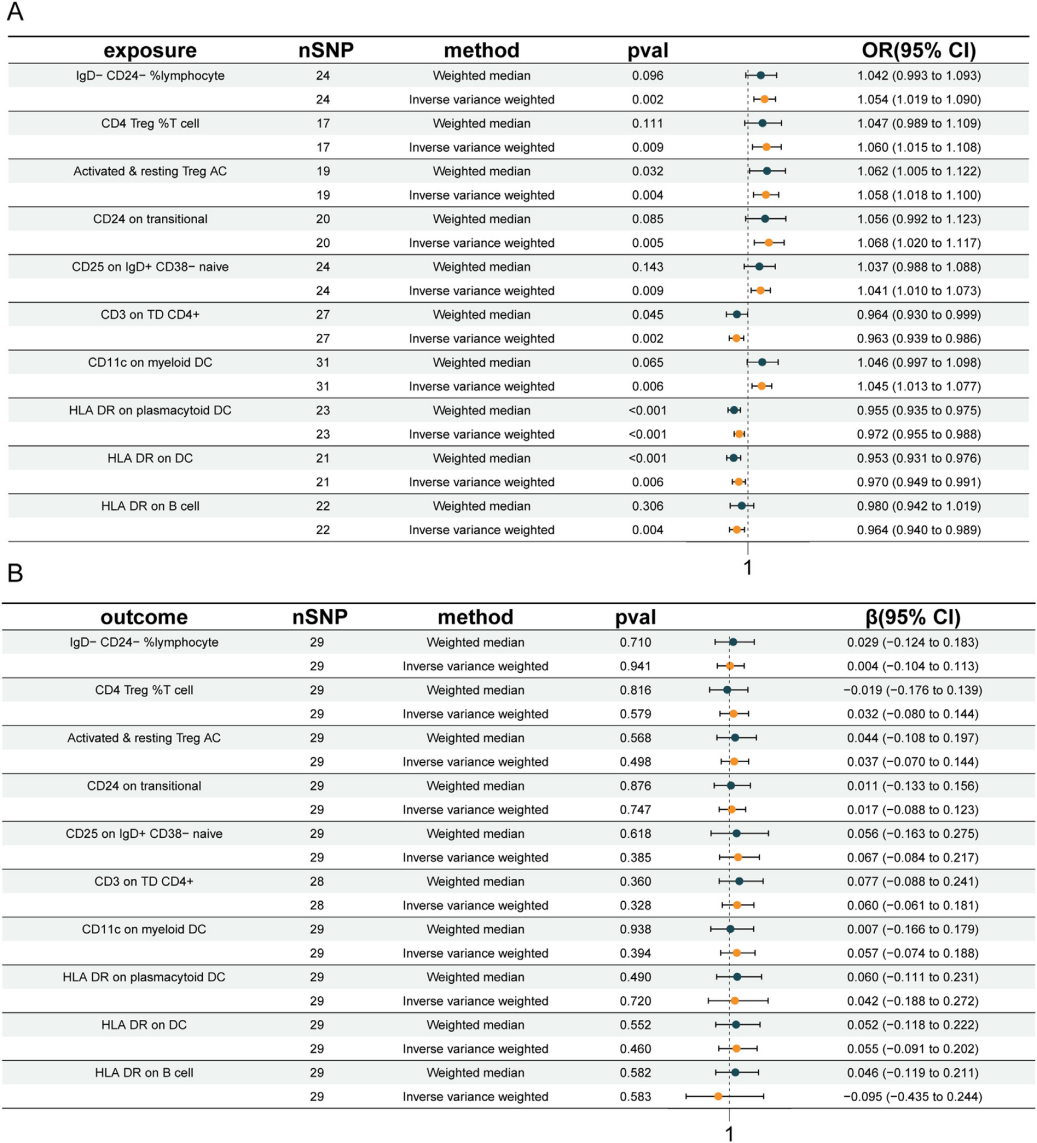
Bidirectional Mendelian randomization of immune cell characteristics and urolithiasis. **(A)** Causal effects of immune cells characteristics on urolithiasis. **(B)** Causal effects of urolithiasis on immune cells characteristics. CI, confidence interval; OR, odds ratio; SNP, single nucleotide polymorphism.

Then, we conducted the reverse MR analysis to explore the causal effects of urolithiasis on immune cell characteristics (Fig. 3B and Table S6). For the criteria used to select significant SNPs for urolithiasis in the reverse MR analysis, we employed SNPs with a mutation frequency (minor allele frequency ≥ 5%) at a linkage disequilibrium threshold *R*^2^ < 0.001 and predicted the exposure significantly at the genetic level (*p* < 5 × 10^−8^). Urolithiasis demonstrated the non-statistical significance of the above 10 immune cell characteristics, indicating the inexistence of reverse causal relationships.

### Screening the causal effect of blood metabolites on urolithiasis

**Figure 4:**
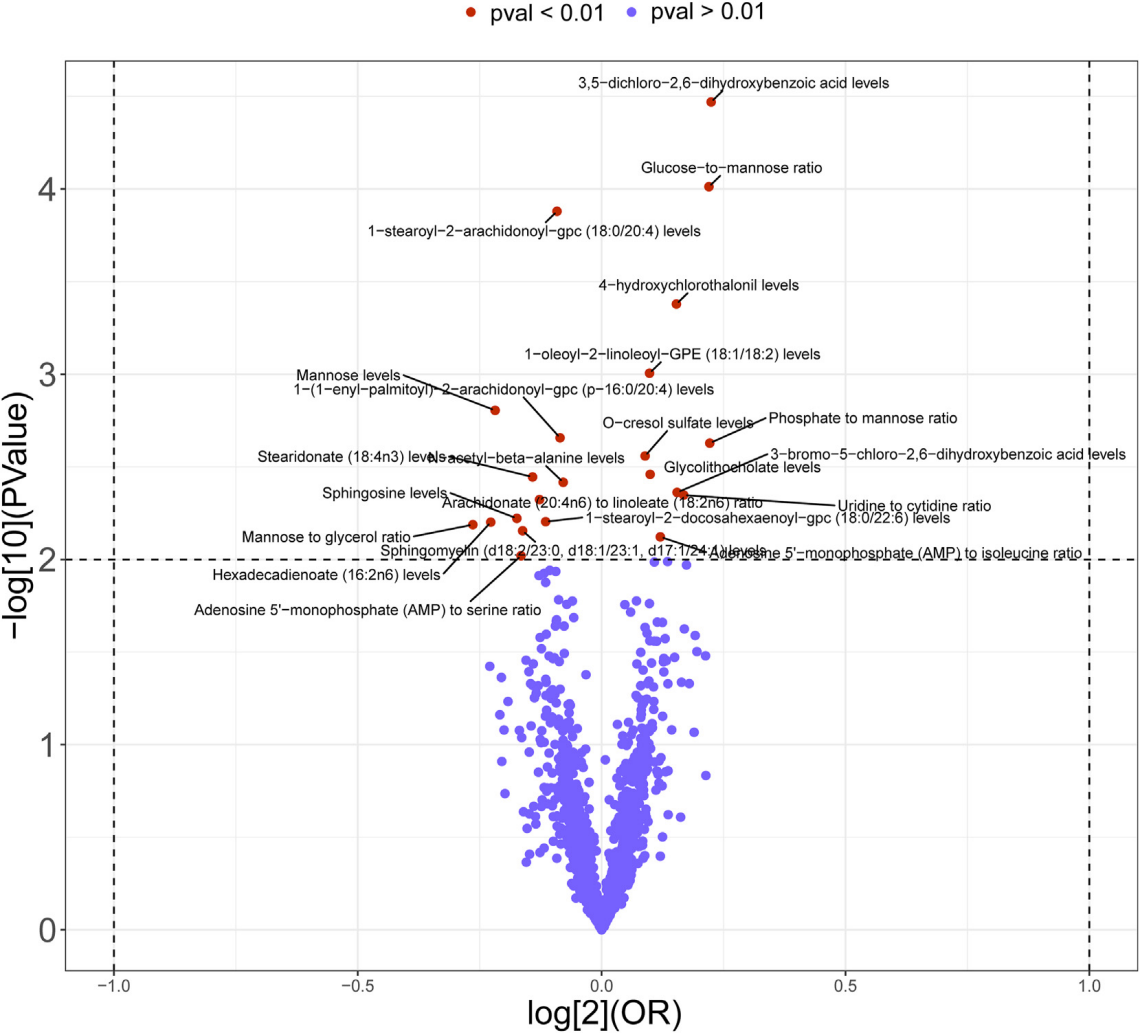
The volcano plot illustrating the link between 1400 blood metabolites and urolithiasis risk. The *X*-axis represents the logarithmic odds ratio (OR) with a base of 2, and the *Y*-axis represents the IVW logarithmic *p*-value with a base of 10. *p* < 0.01 is considered statistically significant.

**Figure 5:**
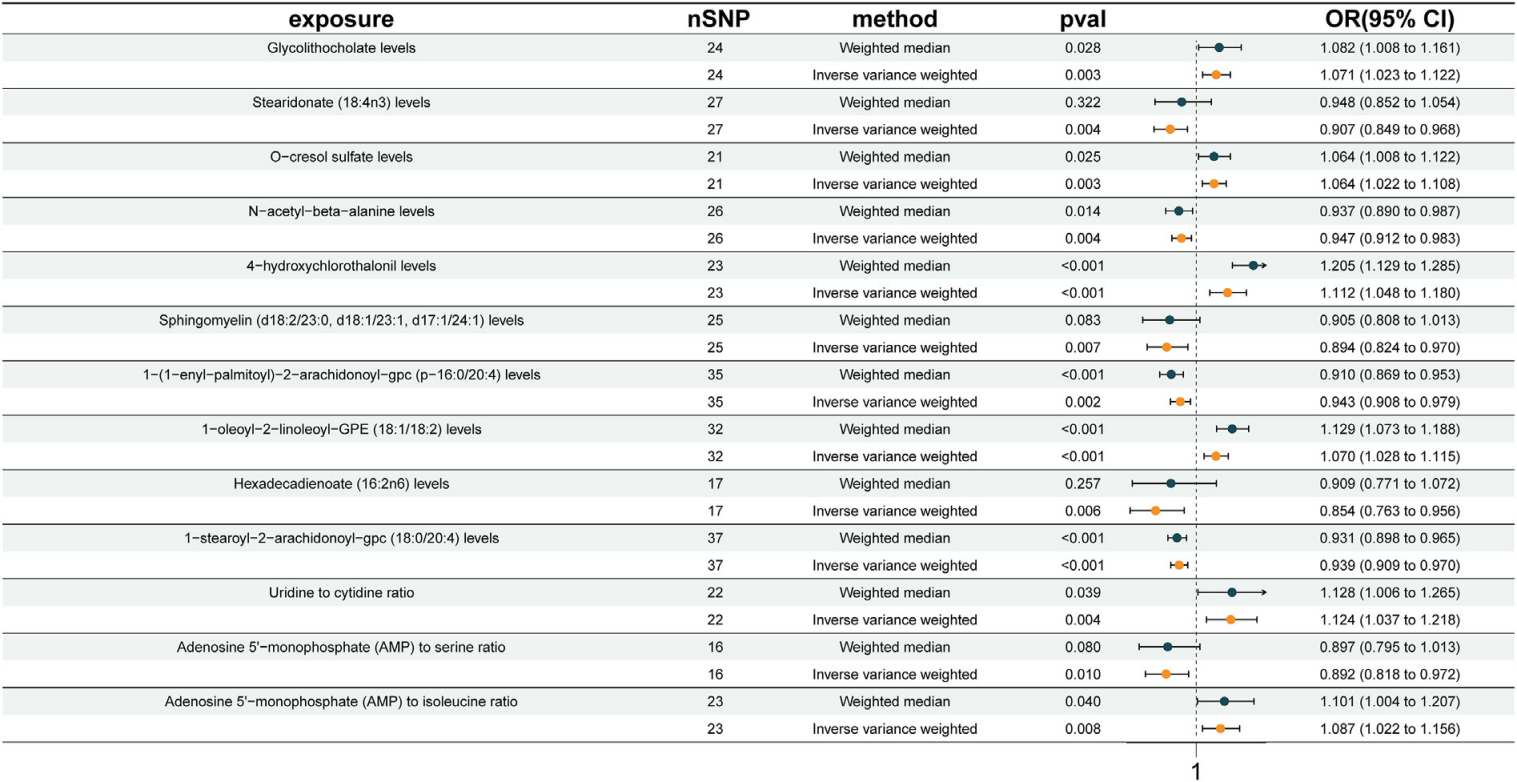
Causal effects of blood metabolites on urolithiasis. CI, confidence interval; OR, odds ratio; SNP, single nucleotide polymorphism.

1400 metabolites and 34,123 independent SNP-component instrumental factors were gathered for this investigation. Table S7 displays an overview of the SNPs that were utilized as genetic tools. The estimated IVs' minimum F-value was more than 10, suggesting that the 1400 metabolites' IVs were reliable for MR analysis. As shown in Figure 4, the volcano plot illustrates the MR analysis results between 1400 blood metabolites and urolithiasis risk. IVW *p*-value < 0.01 was considered statistically significant. Then, we filtered the above results for inconformity using the following standards: i) IVW *p*-value <0.01; ii) Five MR models had consistent direction; iii) Pleiotropy *p*-value <0.05 (Table S8). As shown in Figure 5, 13 metabolites associated with urolithiasis were detected through the IVW method (*p* < 0.01), including 6 lipids, 1 amino acid, 3 carbohydrate, and 3 metabolite ratios. For the metabolites of lipids, one trait was linked with increased urolithiasis risk: Glycolithocholate levels (IVW: OR = 1.071; 95% CI: 1.023, 1.122; *p* = 0.003); five traits were linked with decreased urolithiasis risk: stearidonate (18:4n3) levels (IVW: OR = 0.907; 95% CI: 0.849, 0.968; *p* = 0.004), sphingomyelin (d18:2/23:0, d18:1/23:1, d17:1/24:1) levels (IVW: OR = 0.894; 95% CI: 0.824, 0.970; *p* = 0.007), 1-(1-enyl-palmitoyl)-2-arachidonoyl-gpc (p-16:0/20:4) levels (IVW: OR = 0.943; 95% CI: 0.908, 0.979; *p* = 0.002), hexadecadienoate (16:2n6) levels (IVW: OR = 0.854; 95% CI: 0.763, 0.956; *p* = 0.006), and 1-stearoyl-2-arachidonoyl-gpc (18:0/20:4) levels (IVW: OR = 0.939; 95% CI: 0.909, 0.970; *p* = 0.0001). For the metabolites of amino acids, one trait was linked with increased urolithiasis risk: O-cresol sulfate levels (IVW: OR = 1.064; 95% CI: 1.022, 1.108; *p* = 0.003). For the metabolites of carbohydrate, one trait was linked with decreased urolithiasis risk: N-acetyl-beta-alanine levels (IVW: OR = 0.947; 95% CI: 0.912, 0.983; *p* = 0.004), two traits were linked with increased urolithiasis risk: 4-hydroxychlorothalonil (4-OH-CHT) levels (IVW: OR = 1.112; 95% CI: 1.048, 1.180; *p* = 0.001), and 1-oleoyl-2-linoleoyl-GPE (18:1/18:2) levels (IVW: OR = 1.070; 95% CI: 1.028, 1.115; *p* = 0.001). For the metabolite ratio, two traits were linked with increased urolithiasis risk: uridine to cytidine ratio (IVW: OR = 1.124; 95% CI: 1.037, 1.218; *p* = 0.004) and adenosine 5′-monophosphate to isoleucine ratio (IVW: OR = 1.087; 95% CI: 1.022, 1.156; *p* = 0.008). One trait was linked with decreased urolithiasis risk: adenosine 5′-monophosphate to serine ratio (IVW: OR = 0.892; 95% CI: 0.818, 0.972; *p* = 0.010).

### Investigation of the relationship between immune cell properties and blood metabolites associated with urolithiasis

**Figure 6:**
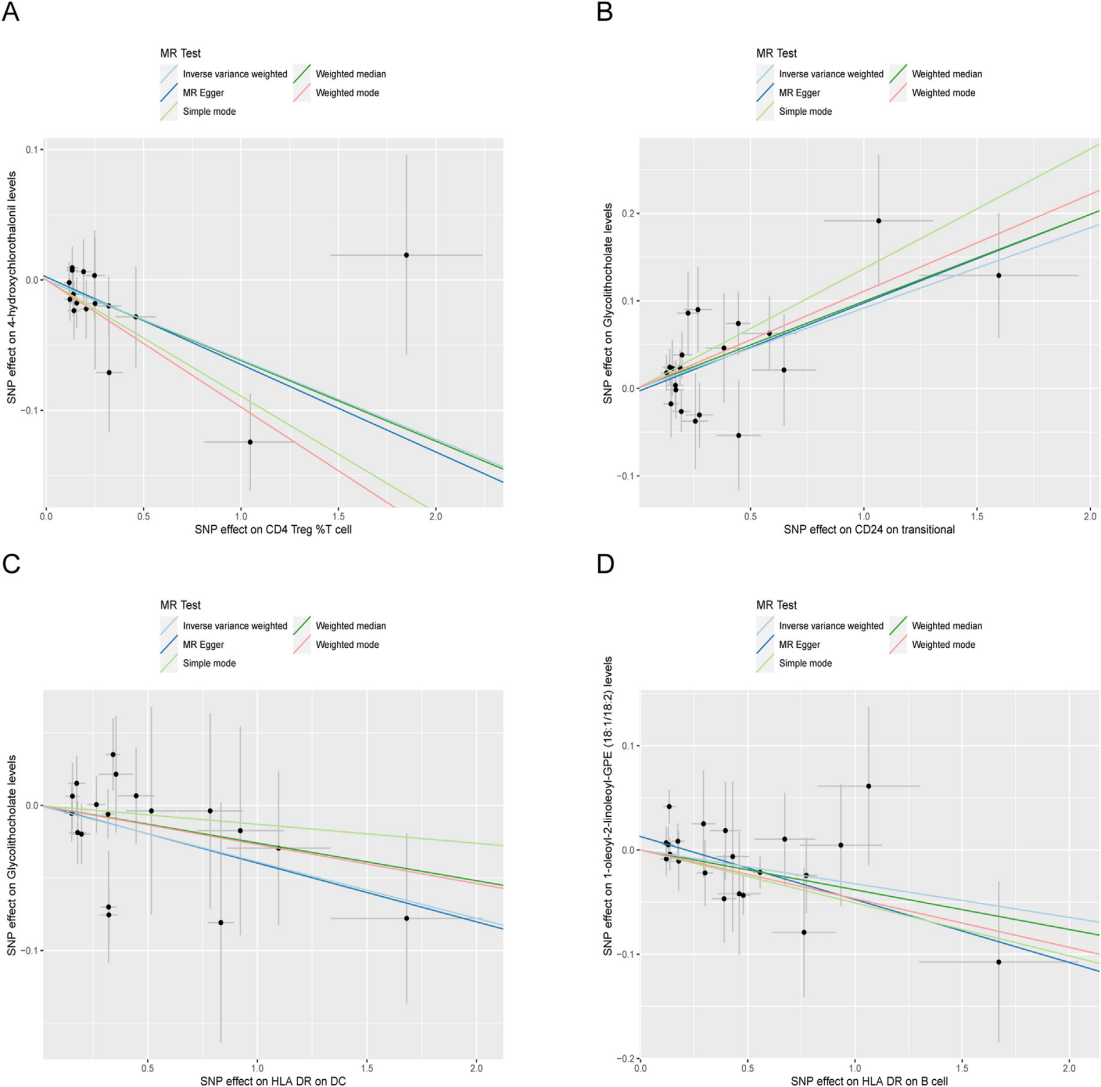
Scatter plots of immune cell characteristics on blood metabolites. **(A)** The negative direction of CD4 Treg %T cell and 4-hydroxychlorothalonil levels. **(B)** The positive direction of CD24 on transitional B cells and glycolithocholate levels. **(C)** The negative direction of HLA DR on DC and glycolithocholate levels. **(D)** The negative direction of HLA DR on B cells and 1-oleoyl-2-linoleoyl-GPE (18:1/18:2) levels.

Based on the screening results, we identified 13 urolithiasis-related blood metabolites. Then, we analyzed the immune cell characteristics and urolithiasis-related blood metabolites using two-sample MR (Table S9). We filtered the above results for inconformity using the following standards: i) IVW *p*-value <0.05; ii) Five MR models had consistent direction; iii) Pleiotropy *p*-value < 0.05. As shown in Figure 6, we found four groups with statistical significance: CD24 on transitional B cells was positive linked with glycolithocholate levels (IVW: *β* = −0.092; 95% CI, 0.045–0.138; *p* = 0.0001), CD4 Treg %T cell was negative associated with 4-OH-CHT levels (IVW: *β* = −0.061; 95% CI: 0.101, −0.021; *p* = 0.02), HLA DR on DC was negative associated with glycolithocholate levels (IVW: *β* = −0.039; 95% CI: 0.077, −0.001; *p* = 0.04), HLA DR on B cell was negative associated with 1-oleoyl-2-linoleoyl-GPE (18:1/18:2) levels (IVW: *β* = −0.032; 95% CI: 0.062, −0.003; *p* = 0.03). Then, we conducted the heterogeneity test (Table S10) and the pleiotropy test (Table S11), which indicated the existence of heterogeneity and pleiotropy.

### Blood metabolites partly mediate the association between immune cell characteristics and urolithiasis

**Figure 7:**
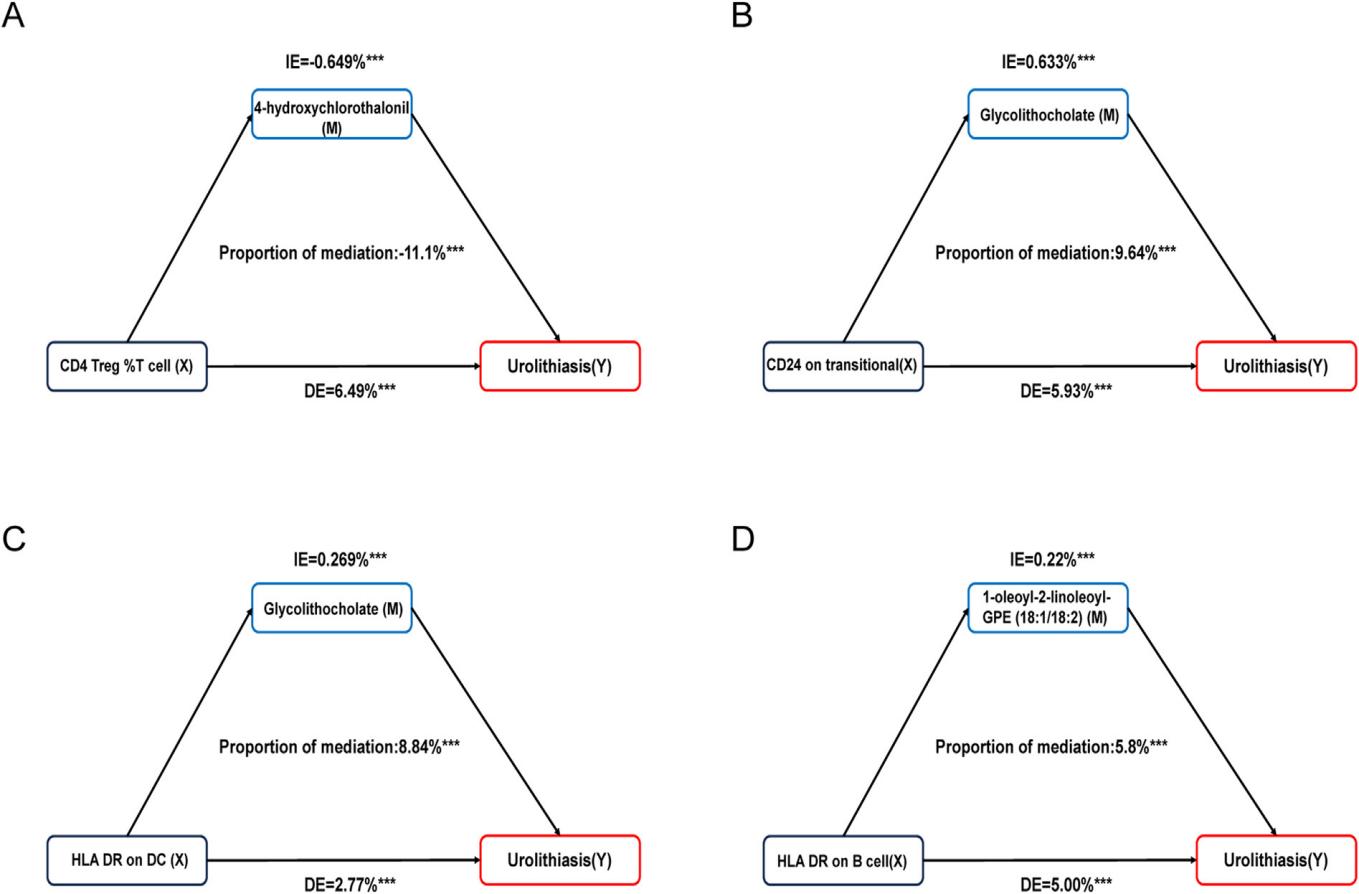
Estimated proportion of the association between immune cell characteristics and urolithiasis mediated by blood metabolites. **(A)** CD4 Treg %T cell mediated by 4-hydroxychlorothalonil. **(B)** CD24 on transitional B cells mediated by glycolithocholate levels. **(C)** HLA DR on DC mediated by glycolithocholate levels. **(D)** HLA DR on B cells mediated by 1-oleoyl-2-linoleoyl-GPE (18:1/18:2). IE, indirect effect; DE, direct effect. ∗∗∗*p* < 0.001.

Mediation analysis was used to evaluate the mediation effect between immune cell characteristics and urolithiasis by blood metabolites (Table S12). We found four group mediation effects with statistical significance, which illustrated the potential pathogenesis of urolithiasis (Fig. 7). First, we found 4-OH-CHT partly negatively mediated the associations between CD4 Treg %T cells and urolithiasis, and the proportions were −11.1%. Then, glycolithocholate levels partly positively mediated the associations between CD24 on transitional B cells and HLA DR on DC with urolithiasis, and the proportions were 9.64% and 8.84%, respectively. Last, 1-oleoyl-2-linoleoyl-GPE (18:1/18:2) partly positively mediated the associations between HLA DR on B cells and urolithiasis, and the proportions were 5.8%.

### Sensitivity analysis

To further confirm the MR causal relationships, we performed pleiotropy and heterogeneity tests (Table S13, 14). No definite indication of pleiotropy was found by the MR-PRESSO study (all *p* > 0.05) (Table S15). Furthermore, no SNP was responsible for the discovered causal relationships, according to the leave-one-out analysis (Fig. S1, 2).

## Discussion

### MR study confirmed the causal associations between immune cell characteristics and urolithiasis

The prevalence of urolithiasis is rising in the United States and across the world, yet little is known about urolithiasis's precise mechanisms.^25^ Despite all this, the genetic effect on stone formation remains significant. Through GWAS and candidate gene studies, it has been shown that a variety of genes and biological mechanisms increase the risk of stone development.^26^ Therefore, we use MR as a genetic epidemiological method to explore possible risk factors for urolithiasis, which is a tool that can effectively verify causal effects, because confounding factors are less likely to have an impact on MR. In this study, we explore the causal relationships and potential mediators between immune cell characteristics and urolithiasis from a genetic perspective.

The development of stones is intimately linked to the immune-inflammatory response. Prior research has demonstrated that proinflammatory cytokine production, immune cell counts, and cellular apoptosis in the tissue of Randall's plaques accelerate the development and progression of stones.^27^ Our finding that B cell traits, Tregs panel, and one trait of CDC panel were linked with increased urolithiasis risk, including IgD-CD24-%lymphocyte, CD24 on transitional B cells, CD25 on IgD^+^ CD38-naïve, CD4 Treg% T cell, activated and resting Treg AC, and CD11c on myeloid DC. However, only CD24 on transitional B cells and CD4 Treg% T cells may promote the formation of stones through blood cell metabolites.

Maintaining immunological homeostasis and preventing autoimmune illnesses requires CD4 Treg %T cells, which are a subset of CD4 T cells that express high levels of the interleukin (IL)-2 receptor α-chain (CD25).^28^, ^29^, ^30^ Williams et al revealed that sustained forkhead box P3 (Foxp3) expression is necessary for mature Treg cells to maintain their suppressor function,^31^ while mice and humans with a loss-of-function mutation in the Foxp3 gene may suffer from disorders manifested by diabetes and cytokine storms.^32^ Similarly, patients with chronic kidney disease often have CD4^+^ Treg dysfunction.^33^ Zhang et al constructed an immune landscape in nephrolithiasis models, which indicates that the inflammatory response induced by CaOx in CD4^+^ T cells and M1 macrophages can promote the formation of stones, revealing the complex relationships between CD4 Treg%T cells and urolithiasis.^34^ CD24 on transitional B cells represents a central developmental stage in B-cell maturation, producing high levels of IL-10, which is considered to be related to chronic inflammatory and autoimmune diseases,^35^^,^^36^ including multiple sclerosis, rheumatoid arthritis, and systemic lupus erythematosus.^37^ Urolithiasis is increasingly recognized as a systemic disease and is associated with systemic disorders, including autoimmune diseases and metabolic syndrome,^38^^,^^39^ renal tubular metabolic acidosis, and hypoparathyroidism.^40^^,^^41^ Based on the above evidence, CD4 Treg% T cells and CD24 on transitional B cells may affect the occurrence and development of stones by affecting the body's autoimmune homeostasis.

Beyond that, we also screened out HLA DR on DC and HLA DR on B cells as protective factors for urolithiasis. The strongest genetic association with autoimmunity is the HLA complex. One class of HLA molecules is MHC class II (HLA DP/DQ/DR) which is predominantly present in professional antigen-presenting cells (dendritic cells, B cells, and macrophages).^42^^,^^43^ Research has shown that HLA is associated with many autoimmune diseases, including autoimmune liver diseases, ankylosing spondylitis, type 1 diabetes mellitus, and autoimmune central nervous system disorders.^44^, ^45^, ^46^ However, in the King's College study, HLA demonstrates its protective effect on autoimmune hepatitis.^47^ Similarly, in adults with AIH, HLA DRB1∗1501 was associated with protection from disease.^48^ Immune cells expressing HLA-DR are expected to become protective predictive markers for urolithiasis, although the mechanisms need further exploration.

### The causal relationships between blood metabolites and urolithiasis were validated by an MR investigation

Obesity, diabetes, hypertension, and metabolic syndrome can all have an impact on the complicated etiology of urolithiasis,^14^ and non-infectious stones are closely associated with metabolic disorders.^49^ Approximately 80% of stones are composed of pure calcium oxalate or mixed with calcium phosphate.^25^ We have identified 13 metabolites related to urolithiasis by MR study, which include 6 lipids, 1 amino acid, 3 carbohydrate, and 3 metabolite ratios. Wherein, glycolithocholate, N-acetyl-beta-alanine, 4-OH-CHT, 1-oleoyl-2-linoleoyl-GPE, the uridine-to-cytidine ratio, and the adenosine-5′-monophosphate-to-isoleucine ratio were linked with increased urolithiasis risk. Stearidonate, sphingomyelin, 1-(1-enyl-palmitoyl)-2-arachidonoyl-gpc, hexadecadienoate, 1-stearoyl-2-arachidonoyl-gpc, and O-cresol sulfate were linked with protective urolithiasis risk.

There is too much evidence to prove that metabolism is closely related to urolithiasis, and investigations into urolithiasis continue to benefit from metabolic screening.^14^ Previous research indicates that hypercalciuria is the most common metabolic abnormality in idiopathic calcium oxalate stone formers.^50^ The formation of calcium oxalate stones is positively correlated with hyperoxalemia, as oxalate can bind to calcium to form calcium oxalate, which is more common in inflammatory bowel disease, small bowel resection, and gastric bypass surgery.^51^ In addition, reducing the intake of fat and dairy products in the diet can reduce the risk of urolithiasis by reducing urinary oxalate excretion.^52^ However, if the intake of fresh fruits, vegetables, and potassium is increased, the risk of stones can be reduced by reducing urate excretion and alkalizing urine.^53^ In summary, metabolism and stones have a very complex interaction relationship, and their specific mechanism needs further clarification.

### MR study confirmed the causal associations between immune cell characteristics and blood metabolites

We investigated the causal relationships between genetically predicted immune cell features and putative mediators that are strongly linked to urolithiasis before doing the mediation analysis. Using two-sample MR, we found four groups with statistical significance, which are mainly focused on the B cell panel, Treg cell panel, CDC panel, and metabolites of lipids and carbohydrates.

Immune and metabolic factors are both high-risk factors for urolithiasis. It has been proven that inflammation damages the kidneys by promoting changes in the body's metabolism, including the formation of CaOx crystals.^54^^,^^55^ Studies have shown that activation of NLRP3 (NLR family pyrin domain containing 3) inflammasomes during acute kidney injury can promote the formation of CaOx crystals, thereby exacerbating acute kidney injury.^55^ After blocking the NLRP3 inflammasome, there was less oxidative damage and higher anti-inflammatory infiltration in the kidneys of experimental mice, which exhibits the potential prospects of immunotherapy in the treatment of urolithiasis.^56^

A study based on the urolithiasis-fecal microbiota transplantation rats' model also revealed the close association between immune regulation, metabolic disorders, and stone formation.^3^^,^^57^ Gut dysbiosis has been proven to correlate with increased endotoxins, especially lipopolysaccharides.^58^ Lipopolysaccharides can cause immunological homeostasis to be disrupted and increase intestinal epithelial permeability by overactivating antigen-presenting cells such as dendritic cells and macrophages, which elevate serum oxalate levels and contribute to the formation of calcium oxalate crystals in the kidneys.^59^^,^^60^

Additionally, recent studies have also demonstrated how the immune system influences metabolites such as oxalate crystals thereby affecting the progression of stones. A study using animal models has shown that M1 macrophages increase the expression of pro-inflammatory chemicals, which facilitates the adherence of CaOx crystals on renal tubular cells and the development of CaOx stones, whereas M2 macrophages have the opposite effect.^11^^,^^61^ The transformation of monocytes into M1 macrophages can be achieved by recognizing CaOx crystals, which is mediated by lipopolysaccharides.^11^^,^^34^ However, the overproduction of reactive oxygen species in monocytes caused by oxalate and CaOx can damage their mitochondria and impair the clearance of crystals in patients with immunological dysfunction.^62^ In summary, the connection between the renal immune system and blood metabolites is intricate and variable, requiring further exploration to clarify their relationship.

### The possible factors mediating immune cells-induced urolithiasis

Finally, we conducted the mediation analysis to clarify the relationship between immune cells, blood metabolites, and urolithiasis. The findings indicated that 4-OH-CHT, glycolithocholate, and 1-oleoyl-2-linoleoyl-GPE might play crucial roles in immune cell-induced urolithiasis.

Aquatic species have been shown to be toxically affected by 4-OH-CHT, a major intermediate product of the breakdown of toxic fungicides.^63^ There are different routes of exposure to 4-OH-CHT for the general population, such as ingestion, respiration, and skin contact.^64^ As of right now, research has shown that exposure to specific chemicals, such as pesticides and herbicides like N,N-Diethyl-meta-toluamide, may raise the risk of developing urolithiasis.^9^^,^^65^ Furthermore, the 4-OH-CHT enhanced pro-inflammatory cytokine production in keratinocytes compared with the photodegraded products, which may also contribute to the formation of urolithiasis.^66^

As one of the bile acids, glycolithocholate participates in the progression of various metabolic diseases, especially metabolic dysfunction-associated steatotic liver disease and intrahepatic cholestasis.^67^^,^^68^ Bile acids may induce inflammatory cytokines, gut microbiota imbalances, and hyperendotoxemia to promote the development of diseases,^69^ and a review indicates that glycolithocholate is related to metabolic-related diseases such as hepatobiliary disorders and diabetes.^70^ Obese patients who undergo gastric bypass surgery (Roux-en-Y) have an increased risk of stones due to disturbed enterohepatic bile circulation.^71^ Existing evidence suggests that there is a definite association between metabolic syndrome and urolithiasis disease, implying a potential connection between glycolithocholate and urolithiasis.^72^

1-oleoyl-2-linoleoyl-GPE is one of the lipid metabolites and their derived signaling molecules, which is regarded as the risk factor for Crohn's disease. However, we have not found any reports on the relationship between 1-oleoyl-2-linoleoyl-GPE and stones, which encourages us to explore more about it.^73^

In summary, our findings suggest a strong relationship between immune cells and blood metabolites in the development of urinary stones, with immune cells either mediating or promoting stone formation and advancement or providing protection through blood metabolites. The specific mechanism needs further exploration.

### Strengths and limitations of this study

In general, our study has several advantages. First, in observational settings, MR research can simulate the costly, laborious, and time-consuming character of randomized controlled trials. Second, MR investigations, compared with other observational studies, are less prone to confounding between exposure and outcome and can prevent the reverse causal effect. Third, the findings were novel. When employed more extensively in clinical practice, the metabolic pathway of blood metabolites in mediating the influence of immune cells on urolithiasis risk may aid in understanding the effect and processes. Certain restrictions and shortcomings are unavoidable. First, although the random effects IVW model is applied to limit the influence of heterogeneity in MR research as much as possible, the results of heterogeneity testing show that there is still some degree of variability among IVs in the investigations. Second, due to the inclusion of only patients of European heritage, there may be limitations to the applicability of our research findings to other demographics. Meanwhile, since MR is a preliminary causal inference based on large data, proving its results requires a more detailed design, so further proof is not so easy.

## Conclusion

This MR research shows the intricate connection between urolithiasis, blood metabolites, and immune responses. The findings emphasize the importance of considering both immune cell features and metabolic factors in understanding the pathogenesis of urolithiasis, offering insights into novel therapeutic targets and diagnostic strategies for this disorder.

## Funding

This research received financial support from various sources including the Innovation and Development Joint Fund of Chongqing 10.13039/501100001809Natural Science Foundation of China (No. CST-B2023NSCQ-LZX0099), Chongqing Science and Health Joint Medical High-end Talent Project (China) (No. 2022GDRC012), Science and Technology Research Program of Chongqing Municipal Education Commission of China (No. KJZD-K202100402), CQMU Program for Youth Innovation in Future Medicine (Chongqing, China) (No. W0073), National Natural Science Foundation of China (82401866), and Special Grants for Postdoctoral Research Projects in Chongqing in 2023 (2023CQBSHTB3134).

## CRediT authorship contribution statement

**Chengcheng Wei:** Data curation, Formal analysis, Methodology, Visualization, Writing – original draft, Writing – review & editing. **Jiattai He:** Data curation, Formal analysis, Methodology, Visualization, Writing – original draft, Writing – review & editing. **Jun Wen:** Data curation, Formal analysis, Visualization, Writing – original draft, Writing – review & editing. **Shunyao Wang:** Visualization, Writing – original draft, Writing – review & editing. **Mengjia Shi:** Visualization, Writing – original draft, Writing – review & editing. **Juan Hu:** Visualization, Writing – original draft, Writing – review & editing. **Huanhuan Tan:** Funding acquisition, Writing – review & editing. **Jinjun Guo:** Funding acquisition, Writing – review & editing. **Xiaosong Li:** Funding acquisition, Writing – review & editing.

## Conflict of interests

The authors declared no conflict of interests.

## Figures and Tables

**figs1:**
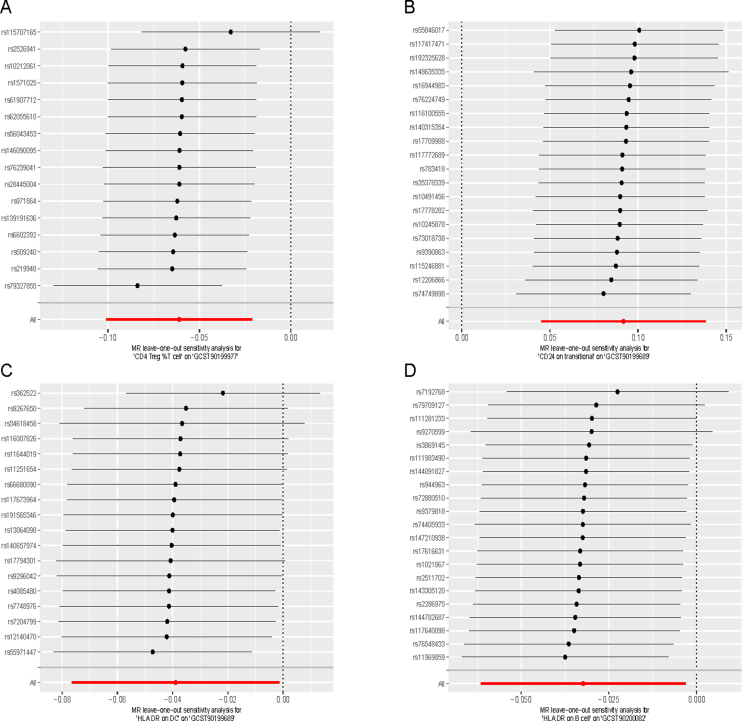


**figs2:**
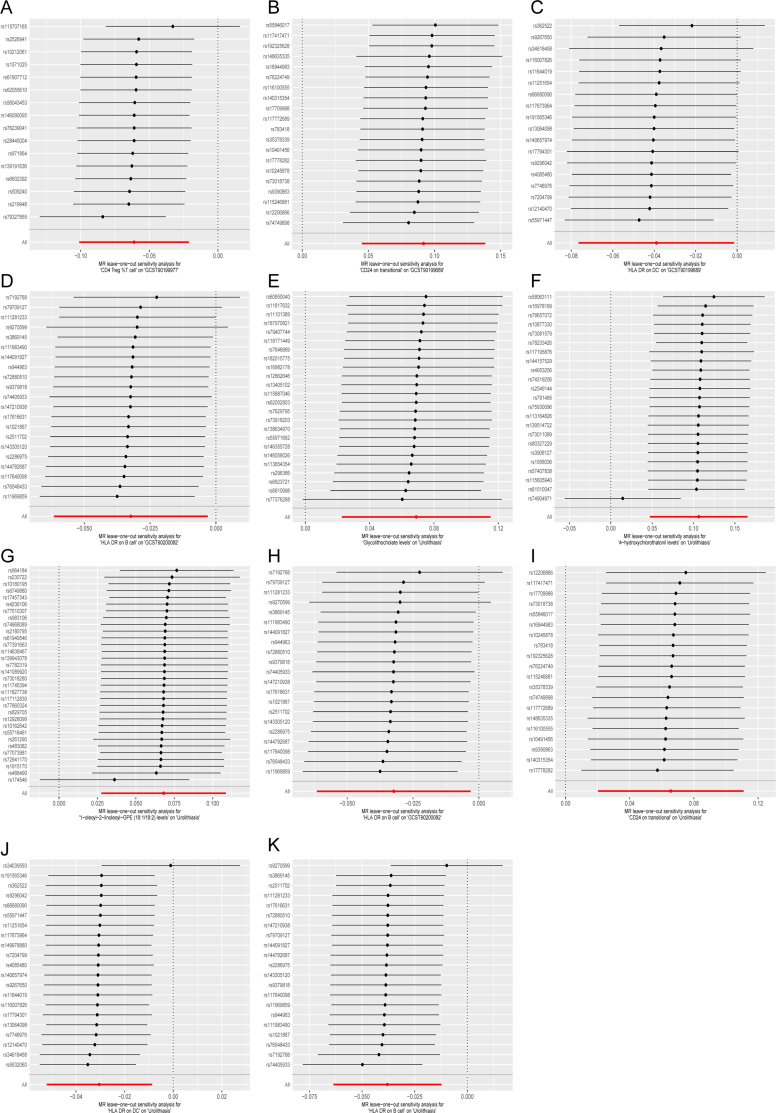


## References

[1] Peerapen P., Thongboonkerd V. (2023). Kidney stone prevention. Adv Nutr.

[2] Tzelves L., Türk C., Skolarikos A. (2021). European association of urology urolithiasis guidelines: where are we going?. Eur Urol Focus.

[3] Hunthai S., Usawachintachit M., Taweevisit M. (2024). Unraveling the role of gut microbiota by fecal microbiota transplantation in rat model of kidney stone disease. Sci Rep.

[4] Sorokin I., Mamoulakis C., Miyazawa K., Rodgers A., Talati J., Lotan Y. (2017). Epidemiology of stone disease across the world. World J Urol.

[5] Raja A., Hekmati Z., Joshi H.B. (2016). How do urinary calculi influence health-related quality of life and patient treatment preference: a systematic review. J Endourol.

[6] Antonelli J.A., Maalouf N.M., Pearle M.S., Lotan Y. (2014). Use of the National Health and Nutrition Examination Survey to calculate the impact of obesity and diabetes on cost and prevalence of urolithiasis in 2030. Eur Urol.

[7] Khan S.R., Pearle M.S., Robertson W.G. (2016). Kidney stones. Nat Rev Dis Prim.

[8] Mehta M., Goldfarb D.S., Nazzal L. (2016). The role of the microbiome in kidney stone formation. Int J Surg.

[9] Wei C., He J., Wei Z. (2023). Association between N, N-diethyl-m-toluamide exposure and the odds of kidney stones in US adults: a population-based study. Front Public Health.

[10] Khan S.R., Canales B.K., Dominguez-Gutierrez P.R. (2021). Randall's plaque and calcium oxalate stone formation: role for immunity and inflammation. Nat Rev Nephrol.

[11] Dominguez-Gutierrez P.R., Kusmartsev S., Canales B.K., Khan S.R. (2018). Calcium oxalate differentiates human monocytes into inflammatory M1 macrophages. Front Immunol.

[12] Kumar P., Yang Z., Lever J.M. (2022). Hydroxyproline stimulates inflammation and reprograms macrophage signaling in a rat kidney stone model. Biochim Biophys Acta, Mol Basis Dis.

[13] Zhang X.Z., Lei X.X., Jiang Y.L. (2023). Application of metabolomics in urolithiasis: the discovery and usage of succinate. Signal Transduct Targeted Ther.

[14] Johri N., Cooper B., Robertson W., Choong S., Rickards D., Unwin R. (2010). An update and practical guide to renal stone management. Nephron Clin Pract.

[15] Daudon M., Jungers P. (2004). Clinical value of crystalluria and quantitative morphoconstitutional analysis of urinary calculi. Nephron Physiol.

[16] Singh P., Enders F.T., Vaughan L.E. (2015). Stone composition among first-time symptomatic kidney stone formers in the community. Mayo Clin Proc.

[17] Abid A., Raza A., Khan A.R. (2023). Primary hyperoxaluria: comprehensive mutation screening of the disease causing genes and spectrum of disease-associated pathogenic variants. Clin Genet.

[18] Negri A.L., Del Valle E.E. (2022). Role of claudins in idiopathic hypercalciuria and renal lithiasis. Int Urol Nephrol.

[19] Messa P., Castellano G., Vettoretti S. (2023). Vitamin D and calcium supplementation and urolithiasis: a controversial and multifaceted relationship. Nutrients.

[20] Smith G.D., Ebrahim S. (2004). Mendelian randomization: prospects, potentials, and limitations. Int J Epidemiol.

[21] Chen J., Chen X., Xie Y., Sun Y., Wang X., Hesketh T. (2021). Irritable bowel syndrome and migraine: evidence from Mendelian randomization analysis in the UK biobank. Expet Rev Gastroenterol Hepatol.

[22] Orrù V., Steri M., Sidore C. (2020). Complex genetic signatures in immune cells underlie autoimmunity and inform therapy. Nat Genet.

[23] Sidore C., Busonero F., Maschio A. (2015). Genome sequencing elucidates Sardinian genetic architecture and augments association analyses for lipid and blood inflammatory markers. Nat Genet.

[24] Zhang T., Chen Y., Li X., Zhang J., Duan L. (2024). Genetic associations and potential mediators between psychiatric disorders and irritable bowel syndrome: a Mendelian randomization study with mediation analysis. Front Psychiatr.

[25] Worcester E.M., Coe F.L. (2010). Clinical practice. Calcium kidney stones. N Engl J Med.

[26] Howles S.A., Thakker R.V. (2020). Genetics of kidney stone disease. Nat Rev Urol.

[27] Taguchi K., Hamamoto S., Okada A. (2017). Genome-wide gene expression profiling of Randall's plaques in calcium oxalate stone formers. J Am Soc Nephrol.

[28] Mishra S., Srinivasan S., Ma C., Zhang N. (2021). CD8^+^ regulatory T cell - a mystery to be revealed. Front Immunol.

[29] Malek T.R., Yu A., Vincek V., Scibelli P., Kong L. (2002). CD4 regulatory T cells prevent lethal autoimmunity in IL-2Rbeta-deficient mice. Implications for the nonredundant function of IL-2. Immunity.

[30] Savage P.A., Klawon D.E.J., Miller C.H. (2020). Regulatory T cell development. Annu Rev Immunol.

[31] Williams L.M., Rudensky A.Y. (2007). Maintenance of the Foxp3-dependent developmental program in mature regulatory T cells requires continued expression of Foxp3. Nat Immunol.

[32] Josefowicz S.Z., Lu L.F., Rudensky A.Y. (2012). Regulatory T cells: mechanisms of differentiation and function. Annu Rev Immunol.

[33] Li Y., Liu X., Wang W. (2018). Low-dose IL-2 expands CD4^+^ regulatory T cells with a suppressive function *in vitro via* the STAT5-dependent pathway in patients with chronic kidney diseases. Ren Fail.

[34] Zhang W., Li L., Zhang T. (2021). An immune atlas of nephrolithiasis: single-cell mass cytometry on SIRT3 knockout and calcium oxalate-induced renal injury. J Immunol Res.

[35] Simon Q., Pers J.O., Cornec D., Le Pottier L., Mageed R.A., Hillion S. (2016). In-depth characterization of CD24^high^CD38^high^ transitional human B cells reveals different regulatory profiles. J Allergy Clin Immunol.

[36] Anolik J.H., Barnard J., Owen T. (2007). Delayed memory B cell recovery in peripheral blood and lymphoid tissue in systemic lupus erythematosus after B cell depletion therapy. Arthritis Rheum.

[37] Zhou Y., Zhang Y., Han J., Yang M., Zhu J., Jin T. (2020). Transitional B cells involved in autoimmunity and their impact on neuroimmunological diseases. J Transl Med.

[38] Shastri S., Patel J., Sambandam K.K., Lederer E.D. (2023). Kidney stone pathophysiology, evaluation and management: core curriculum 2023. Am J Kidney Dis.

[39] Ilzkovitz M., Kayembe E.E., Geers C., Pozdzik A. (2022). Kidney stones, proteinuria and renal tubular metabolic acidosis: what is the link?. Healthcare.

[40] Clarke B.L., Brown E.M., Collins M.T. (2016). Epidemiology and diagnosis of hypoparathyroidism. J Clin Endocrinol Metab.

[41] Wagner C.A., Unwin R., Lopez-Garcia S.C., Kleta R., Bockenhauer D., Walsh S. (2023). The pathophysiology of distal renal tubular acidosis. Nat Rev Nephrol.

[42] Pishesha N., Harmand T.J., Ploegh H.L. (2022). A guide to antigen processing and presentation. Nat Rev Immunol.

[43] Mack C.L. (2022). HLA Associations in pediatric autoimmune liver diseases: current state and future research initiatives. Front Immunol.

[44] Zhai Y., Chen L., Zhao Q. (2023). Cysteine carboxyethylation generates neoantigens to induce HLA-restricted autoimmunity. Science.

[45] Ilonen J., Lempainen J., Veijola R. (2019). The heterogeneous pathogenesis of type 1 diabetes mellitus. Nat Rev Endocrinol.

[46] Ramanathan S., Brilot F., Irani S.R., Dale R.C. (2023). Origins and immunopathogenesis of autoimmune central nervous system disorders. Nat Rev Neurol.

[47] Ma Y., Su H., Yuksel M. (2021). Human leukocyte antigen profile predicts severity of autoimmune liver disease in children of European ancestry. Hepatology.

[48] Donaldson P.T. (2004). Genetics of liver disease: immunogenetics and disease pathogenesis. Gut.

[49] Szymanski K.M., Misseri R., Whittam B. (2016). Bladder stones after bladder augmentation are not what they seem. J Pediatr Urol.

[50] Curhan G.C., Willett W.C., Speizer F.E., Stampfer M.J. (2001). Twenty-four-hour urine chemistries and the risk of kidney stones among women and men. Kidney Int.

[51] Dussol B., Verdier J.M., Le Goff J.M., Berthezene P., Berland Y. (2007). Artificial neural networks for assessing the risk factors for urinary calcium stones according to gender and family history of stone. Scand J Urol Nephrol.

[52] Andersson H., Jagenburg R. (1974). Fat-reduced diet in the treatment of hyperoxaluria in patients with ileopathy. Gut.

[53] Taylor E.N., Fung T.T., Curhan G.C. (2009). DASH-style diet associates with reduced risk for kidney stones. J Am Soc Nephrol.

[54] Sun X.Y., Gan Q.Z., Ouyang J.M. (2015). Calcium oxalate toxicity in renal epithelial cells: the mediation of crystal size on cell death mode. Cell Death Dis.

[55] Mulay S.R., Evan A., Anders H.J. (2014). Molecular mechanisms of crystal-related kidney inflammation and injury. Implications for cholesterol embolism, crystalline nephropathies and kidney stone disease. Nephrol Dial Transplant.

[56] Dominguez-Gutierrez P.R., Kwenda E.P., Khan S.R., Canales B.K. (2020). Immunotherapy for stone disease. Curr Opin Urol.

[57] Mishra S.P., Wang B., Jain S. (2023). A mechanism by which gut microbiota elevates permeability and inflammation in obese/diabetic mice and human gut. Gut.

[58] Lukiw W.J. (2016). *Bacteroides fragilis* lipopolysaccharide and inflammatory signaling in Alzheimer's disease. Front Microbiol.

[59] de Vos W.M., Tilg H., Van Hul M., Cani P.D. (2022). Gut microbiome and health: mechanistic insights. Gut.

[60] Jiang Z., Asplin J.R., Evan A.P. (2006). Calcium oxalate urolithiasis in mice lacking anion transporter Slc26a6. Nat Genet.

[61] Taguchi K., Okada A., Hamamoto S. (2016). M1/M2-macrophage phenotypes regulate renal calcium oxalate crystal development. Sci Rep.

[62] Patel M., Yarlagadda V., Adedoyin O. (2018). Oxalate induces mitochondrial dysfunction and disrupts redox homeostasis in a human monocyte derived cell line. Redox Biol.

[63] Kwon J.W., Armbrust K.L. (2006). Degradation of chlorothalonil in irradiated water/sediment systems. J Agric Food Chem.

[64] Kazos E.A., Nanos C.G., Stalikas C.D., Konidari C.N. (2008). Simultaneous determination of chlorothalonil and its metabolite 4-hydroxychlorothalonil in greenhouse air: dissipation process of chlorothalonil. Chemosphere.

[65] Mao W., Hu Q., Chen S. (2021). Polyfluoroalkyl chemicals and the risk of kidney stones in US adults: a population-based study. Ecotoxicol Environ Saf.

[66] Xu W., Vebrosky E.N., Armbrust K.L. (2020). Potential toxic effects of 4-OH-chlorothalonil and photodegradation product on human skin health. J Hazard Mater.

[67] Lai J., Luo L., Zhou T., Feng X., Ye J., Zhong B. (2023). Alterations in circulating bile acids in metabolic dysfunction-associated steatotic liver disease: a systematic review and meta-analysis. Biomolecules.

[68] Yousef I.M., Tuchweber B., Vonk R.J., Massé D., Audet M., Roy C.C. (1981). Lithocholate cholestasis: sulfated glycolithocholate-induced intrahepatic cholestasis in rats. Gastroenterology.

[69] Allen K., Jaeschke H., Copple B.L. (2011). Bile acids induce inflammatory genes in hepatocytes: a novel mechanism of inflammation during obstructive cholestasis. Am J Pathol.

[70] Jia Y., Yang X., Wilson L.M. (2022). Diet-related and gut-derived metabolites and health outcomes: a scoping review. Metabolites.

[71] Sinha M.K., Collazo-Clavell M.L., Rule A. (2007). Hyperoxaluric nephrolithiasis is a complication of Roux-en-Y gastric bypass surgery. Kidney Int.

[72] Wong Y., Cook P., Roderick P., Somani B.K. (2016). Metabolic syndrome and kidney stone disease: a systematic review of literature. J Endourol.

[73] Di'Narzo A.F., Houten S.M., Kosoy R. (2022). Integrative analysis of the inflammatory bowel disease serum metabolome improves our understanding of genetic etiology and points to novel putative therapeutic targets. Gastroenterology.

